# Editorial: The application of phytohormones in plant biotechnology for sustainable agriculture

**DOI:** 10.3389/fpls.2024.1382055

**Published:** 2024-03-05

**Authors:** Nqobile A. Masondo, Shubhpriya Gupta, Mack Moyo, Adeyemi O. Aremu

**Affiliations:** ^1^ Agricultural Research Council – Vegetable Industrial and Medicinal Plants, Pretoria, South Africa; ^2^ Laboratory of Growth Regulators, Faculty of Science, Palacký University & Institute of Experimental Botany AS CR, Olomouc, Czechia; ^3^ Research Centre for Plant Growth and Development, School of Life Sciences, University of KwaZulu-Natal Pietermaritzburg, Scottsville, South Africa; ^4^ Department of Horticulture, Faculty of Applied Sciences, Durban University of Technology, Durban, South Africa; ^5^ School of Life Sciences, College of Agriculture, Engineering and Science, University of KwaZulu-Natal, Durban, South Africa; ^6^ Indigenous Knowledge Systems Centre, Faculty of Natural and Agricultural Sciences, North-West University, Mmabatho, South Africa

**Keywords:** auxins, biotic stress, cytokinins, flowering, phytochemicals, plant growth regulators

The Sustainable Development Goals (SDGs) adopted by the United Nations Member States in 2015 recognize the need for sustainable agriculture, which will benefit the livelihood of humanity and protect the environment. The impact of climate change (SDG 17) and the associated environmental factors remain a concern in agriculture as the demand for food, feed, fiber, and biofuel production continues to intensify. Globally, the application of modern technologies in agriculture such as precision farming techniques (e.g., GPS-guided tractors, drones, and sensors), biotechnology (including genetic engineering and molecular breeding), artificial intelligence (AI), and robotics is imperative as diverse research approaches in the past have contributed to the development of stress-tolerant, high-yielding, and nutritionally rich crop cultivars (Abiri et al., 2023; Ivezić et al., 2023). For instance, biotechnological systems such as the use of phytohormones in sustaining plant productivity have shown remarkable potential in the agricultural productivity. Phytohormones, often regarded as plant growth regulators (PGRs), are key signaling molecules that modulate plant physiological and biochemical processes under favorable and unfavorable conditions (EL Sabagh et al., 2022). These diverse phytohormones [e.g., auxins, cytokinins (CKs), ethylene, gibberellins, brassinosteroids (BRs), jasmonates (JAs), and strigolactones] could be essential metabolic engineering targets that when precisely (up/down) regulated have the potential to improve plant growth and the stress-tolerant index in crops (Wani et al., 2016). Therefore, understanding the interaction of phytohormones in terms of their signaling networks and mechanisms of action is fundamental for agriculture to recognize their multi-faceted role in crop production. The current Research Topic “The Application of Phytohormones in Plant Biotechnology for Sustainable Agriculture” explored the role and mechanisms of action of phytohormones (including synthetic and natural ones) in plants, addressing their physiology, molecular biology, and secondary metabolite production.

The six articles published in the current Research Topic focused on the role of exogenously applied phytohormones [such as JAs, BRs, naphthaleneacetic acid (NAA), N-(2-chloro- 4-pyridyl)-N’-phenylurea (CPPU), and ethephon)] and other growth-inducing arbuscular mycorrhizal fungi (AMF) on plant growth and development (Sohn et al.; Zhu et al.), sex determination (Wu et al.), seed yield (Xie et al.), and biotic (Li et al.) and abiotic stresses (Marková et al.). The possible mechanisms of action of phytohormones at different developmental stages in plants were discussed in terms of antioxidant enzymatic activity, gene expression, endogenous phytohormones, and secondary metabolite regulation. In a review by Sohn et al., the authors provided a comprehensive update on the role of JAs during growth and development and in the elicitation of bioactive compounds (e.g. anthocyanins, alkaloids, coumestans, flavonoids, polyphenols, and triterpenoids) in medicinal plants. JAs act as key regulators of seed germination, floral development, and leaf senescence in plants throughout their developmental stages. The application of JAs in eliciting metabolites has been explored using systems such as cell suspension cultures, callus cultures, and hairy and adventitious root cultures. The authors concluded that JA-elicited phytochemical metabolism machinery often entails several transcription factors including APETALA2/ETHYLENE RESPONSIVE FACTORs (AP2/ERFs), WRKYs, basic helix–loop–helix (bHLHs), and myeloblastosis viral oncogene homolog (R2R3-MYBs). However, these researchers highlighted the need for research on the transcriptional factors of medicinal orphan crops as these represent a gold mine for novel bioactive compounds with industrial applications. Tree species with medicinal benefits are known to accumulate secondary metabolites in their organs (e.g., trunk and roots) that promote the formation of heartwood. It is generally known that heartwood formation can be facilitated with the use of different approaches, such as biotic and abiotic stresses, and the use of phytohormones. Using different PGRs including ethephon, Zhu et al. optimized the formation mechanism and the induction of heartwood in *Dalbergia odorifera* (a rare and endangered tree species). The findings from the study revealed that ethephon triggered a significant increase in endogenous ethylene content, enhanced overall metabolic activity, and upregulated the activities of key enzymes, such as chalcone isomerase (CHI) and terpene synthase (TPS), that are involved in the synthesis of phytochemicals in *Dalbergia odorifera*.

*Castanea henryi*, commonly known as Henry’s chestnut or Chinese chinquapin, is an economically valuable timber and chestnut tree with great economic value. However, its nut yield is hindered by the excessive formation of male flowers relative to female flowers. As a means of improving the yield of *C*. *henryi*, male flowers were converted to female flowers by exogenous application of a CK compound, N-(2-chloro- 4-pyridyl)-N’-phenylurea (CPPU) (Wu et al.). Findings from the study demonstrated that the transformational development of the pistil primordium and stamen primordium occurred at stage 3 (9 days after the last CK treatment) of CPPU application. They also revealed the critical role of signaling genes, such as transcription factors (TFs) WRKY47, ERF021, and MYB4, and floral organ identity genes, in sex determination in *C*. *henryi*. Thus, application of CPPU could potentially solve the issue of having an imbalanced ratio of male to female flowers in *C*. *henryi* by instigating a transformational mechanism.

Phytohormones play a crucial role in seed formation and seed yield. In a study by Xie et al., the authors investigated the effect of phosphorus (P) and NAA application on the physiological and biochemical response of flax (*Linum usitatissimum* L.) for effective agronomic management practices. The combined effect of P and NAA applied at optimum concentrations of 67.5 kg P_2_O_5_ ha^–1^ and 20 mg NAA L^–1^ increased sucrose phosphate synthase (SPS) and Rubisco activities, nitrogen (N) and P content at flowering stage and maturity, the assimilation and translocation of N and P during post-flowering, capsules per plant, and seed yield. The observable increase in SPS and Rubisco activities was attributed to the relative expression of *Linum usitatissimum* sucrose phosphate synthase (*LuSPS1*, *LuSPS2*, *LuSPS3*, *LuSPS4*) and *LuRubisco*. Based on these results, the authors recommended the combination of P and NAA as an effective agronomic management practice for increasing the seed yield of flax for maximum crop productivity.

Phytohormones are commonly applied in agriculture to minimize plant susceptibility to biotic and abiotic stress while improving crop productivity. Disease-suppressing treatments such as AMF and β-aminobutyric acid (BABA) elicitors are beneficial in agriculture as they enhance plant resistance to biotic stressors. They trigger the biosynthesis of secondary metabolites and enzymes that induce plant resistance to diseases. The work by Li et al. reported on the synergistic effect of BABA and AMF in inducing disease resistance in tobacco plants infected with *Phytophthora nicotianae*, which causes tobacco black shank (TBS) disease. The joint application of BABA and AMF was beneficial as it increased plant photosynthesis, antioxidant enzymes [superoxide dismutase (SOD), catalase (CAT), peroxidase (POD), and ascorbate peroxidase (APX)], the *Phytophthora nicotianae* resistance (*Ph*) gene, and phytochemicals (phenolics and flavonoids), which enhanced disease resistance in tobacco plants. Brassinolide [24-epibrassinolide (*epi*BL)], a biologically active BR, was exogenously applied in maize plants exposed to drought stress (Marková et al.). Amongst the factors that were examined, there was accelerated senescence in older leaves (depicted by the decline in chlorophyll content and the reduction in primary photosynthetic parameters) relative to younger leaves that displayed high levels of proline content when *epi*BL was applied in plants. The impact of drought stress on maize plants (young and old leaves) was further noted in the marked differences of endogenous BRs (C_27_, C_28_-and C_29_-BRs). In this case, drought stress downregulated the biosynthetic pathway associated with C_28_-BRs and C_29_-BRs in older leaves, whereas in younger leaves, the main downregulated pathway was C_29_-BRs.

In conclusion, this Research Topic on the application and diverse roles of phytohormones in plant biotechnology for sustainable agriculture has revealed promising breakthroughs in enhancing plant growth, increased yields, and stress mitigation. The articles in this Research Topic have contributed toward the elucidation of the key genes and biosynthetic pathways involved in the physiological mechanisms of plant growth, development, and survival. The new insights into phytohormonal interactions open the door to innovative, eco-friendly solutions to meet global agricultural challenges.

## Author contributions

NM: Conceptualization, Writing – original draft, Writing – review & editing. SG: Writing – review & editing. MM: Writing – review & editing. AA: Conceptualization, Writing – original draft, Writing – review & editing.
